# Nursing care for a patient with syringomyelia complicated by bacterial pneumonia and severe pressure injury: a case report

**DOI:** 10.3389/fmed.2026.1784074

**Published:** 2026-03-24

**Authors:** Yang Wang, Mengyao Wang, Yinhu Tan, Yu Gao, Wenhui Guo, Xiao Feng, Xiang Jia, Qinghuai Yu, Xiuling Zhou

**Affiliations:** 1 School of Nursing, Changchun University of Chinese Medicine, Changchun, Jilin, China; 2 Department of Surgery, Affiliated Hospital of Changchun University of Chinese Medicine, Changchun, Jilin, China

**Keywords:** bacterial pneumonia, infection, nursing, pressure injury, syringomyelia

## Abstract

This study reviews and summarizes the comprehensive nursing process of a patient with a 23-year history of syringomyelia complicated by bacterial pneumonia and pressure injury, with a particular focus on the prevention and control of pneumonia-related infection and pressure injury. Initial health assessment determined that the primary nursing objectives were infection control and correction of malnutrition. During the nursing process, a comprehensive and integrated nursing intervention combining Chinese and Western medicine was implemented, emphasizing three key aspects: prevention and control of respiratory infections, prevention and control of pressure injuries, and nutritional support and complication management. With multidisciplinary pressure injury management and appropriate antibiotic therapy, the patient’s low-grade fever resolved, infection markers decreased, and overall condition improved without secondary infections. The patient’s condition stabilized on the 13th day of hospitalization, and he was successfully discharged and followed up according to changes in his condition. This case highlights the challenges in nursing patients with syringomyelia complicated by multiple comorbidities and underscores the necessity of multidisciplinary collaboration. The coordinated efforts among different specialties contributed to the successful management of this complex case and provide clear clinical guidance for improving the overall quality of care in similar patients.

## 1 Introduction

Syringomyelia is a chronic progressive neurological disorder caused by the formation of cavities within the central canal of the spinal cord or adjacent spinal cord parenchyma (1). Clinically, it often manifests as upper limb muscle atrophy, sensory disturbances, and neurogenic functional impairments. The prevalence of syringomyelia ranges from 9 to 133 per 100,000 in the general population (2). The disease course is complex, particularly in secondary cases, which may develop insidiously and gradually manifest decades after spinal cord injury, with subtle progression that is easily overlooked. MRI-confirmed syringomyelia occurs in approximately 21–28% of patients with spinal cord injury, of whom only 1–9% are symptomatic (2). In patients with spinal cord injury, infection remains an important cause of mortality, and pneumonia and pressure injuries may increase the risk of infectious complications such as septicemia. Pressure injuries are considered the most common secondary complication during hospitalization and rehabilitation (3). Toader et al. (4) reported that pain is a primary symptom of syringomyelia. Early manifestations include limb numbness, paresthesia, diminished or absent sensation to touch, and reduced sensitivity to pain and pressure stimuli, which may progress to segmental dissociated sensory loss. With disease progression, sensitivity to pain and pressure stimuli decreases, predisposing patients to pressure injuries and hypostatic pneumonia. Therefore, a comprehensive understanding of the long-term evolution of this disease is essential for effectively preventing and managing potential late-stage complications, optimizing therapeutic strategies, and improving overall nursing care quality.

Pneumonia is a common and clinically significant respiratory complication in patients with syringomyelia, particularly among those with severe neurological impairment and reduced cough effectiveness (5). When pneumonia coexists, prolonged bed rest or the supine position is often required for respiratory therapy and airway management, thereby increasing pressure and shear forces over bony prominences such as the sacrum, heels, and occiput, which elevates the risk of pressure injuries (3). Neurological deficits and mobility limitations associated with syringomyelia further complicate airway management and sputum clearance (2). When these conditions coexist with malnutrition and systemic inflammatory response, standard nursing protocols become insufficient, and care complexity increases substantially. Existing studies have primarily focused on respiratory support and airway clearance techniques in patients with pneumonia, or on staging management and wound interventions for pressure injuries, whereas reports on comprehensive nursing strategies addressing the coexistence of pneumonia, pressure injury, malnutrition, and other high-risk factors remain relatively limited.

In April 2025, our hospital’s Pulmonary Oncology Department admitted a patient with a 23-year history of syringomyelia complicated by bacterial pneumonia and infected pressure injury and severe malnutrition. The patient also presented with severe anemia, hypoalbuminemia, and systemic inflammatory response syndrome. Nursing assessment identified systemic inflammatory response syndrome and pressure injury as primary nursing issues. Consequently, infection prevention and control became critical, with nursing interventions focusing on three key areas: respiratory infection prevention and control, pressure injury prevention and control, as well as nutritional support and management of complications. After treatment, the patient’s body temperature returned to normal, cough and sputum production were significantly reduced, chest tightness and fatigue improved, inflammatory markers decreased, vital signs remained stable, and the wound area decreased with reduced exudation. The patient’s condition stabilized on the 13th day after admission, leading to a successful discharge with follow-up scheduled based on clinical changes. The detailed nursing process is reported as follows:

## 2 Case description

### 2.1 General information

A 68-year-old female patient was admitted on April 8, 2025, presenting with recurrent fever for 3 days, worsening fatigue for 1 day, after accidental exposure to cold 3 days prior, followed by fever, cough, and chest tightness, with a maximum temperature of approximately 101.3 °F, accompanied by a small amount of yellow sputum and exertional dyspnea that was not significantly relieved by rest; fatigue markedly worsened 1 day before admission, accompanied by decreased mental status. Admission vital signs were as follows: temperature 97.7 °F, respiratory rate 20 breaths/min, heart rate 78 beats/min, and blood pressure 122/76 mmHg. Physical examination revealed scattered moist rales in both lungs. Cardiac examination showed a regular rhythm with muffled heart sounds, without pathological murmurs, pericardial friction rub, or palpable thrills. The patient was lethargic, in a state of mild stupor, with marked fatigue and poor sleep. Skin examination showed an infected pressure injury on the back, presenting as a superficial second-degree ulcer with a diameter of approximately 10 cm. Laboratory findings showed: hemoglobin level of 62.00 g/L, indicating severe anemia; albumin level of 30.5 g/L, indicating hypoalbuminemia; D-dimer significantly elevated to 3,234 ng/mL; lymphocyte percentage 11.80%, lymphocyte count 0.44 × 10^9^/L, below normal range; C-reactive protein elevated to 11.71 mg/L in whole blood; serum albumin decreased to 30.5 g/L; erythrocyte sedimentation rate 51 mm/h, above normal range, indicating a heightened inflammatory response. Past medical history: 23-year history of syringomyelia causing lower limb mobility impairment and urinary/fecal incontinence; over 5-year history of ischemic heart disease; history of compression fractures at T11 and L2 vertebral bodies. Regular lifestyle habits; no drug allergies; no family history of diabetes, hypertension, or hereditary diseases. Based on the clinical presentation, the patient was ultimately diagnosed with bacterial pneumonia, multiple pulmonary nodules, syringomyelia, ischemic heart disease (NYHA Class II), an infected pressure injury, severe anemia, and hypoalbuminemia.

Upon admission, the patient received antimicrobial therapy, expectorant treatment, oxygen therapy, and symptomatic supportive care. A multidisciplinary consultation was conducted with the involvement of the Departments of Nutrition, Dermatology, Hepatology and Gastroenterology, Gastroenterology, and Spine Surgery, as well as a Wound, Ostomy, and Continence (WOC) nurse. The WOC nurse’s initial consultation diagnosed a superficial Grade II pressure injury, recommending local cleanliness, application of Jindi Shengji Ointment (a traditional Chinese herbal ointment for wound healing), and enhanced nutrition. Given increased wound exudate on April 11 and culture results indicating *Escherichia coli* infection, the primary treatment plan per clinical guidelines was intravenous ceftriaxone sodium. The WOC nurse adjusted nursing interventions to include daily wound cleansing, assessment of exudate volume and healing progress, and dressing changes guided by the principle of moist wound healing. To improve nutritional status, short-peptide enteral nutrition was administered, with monitoring of serum albumin levels and gradual escalation of caloric intake.

### 2.2 Treatment process and outcomes

Table 1 details the comprehensive treatment process from admission to discharge after 13 days of hospitalization, following disease stabilization and infection control.

**Table 1:** Treatment process and outcomes.

Time	Key clinical findings	Major interventions	Responses/outcome
Admission day	Admission diagnosis: Bacterial pneumonia; syringomyelia; severe anemia (Hb 62.0 g/L↓); hypoalbuminemia (Alb 30.5 g/L↓); superficial Stage II pressure injury on the back (~10 cm, ulcerated); markedly elevated D-dimer (3,234 ng/mL↑).	Level I nursing care; intermittent low-flow oxygen; alternating-pressure mattress; oral heat-clearing and mood-relieving TCM granules.	Condition stable; baseline labs obtained
Days 2–3 of admission	Fever spike to 38.5°C; worsening fatigue; increased wound exudate.	IV albumin 10 g/day (April 9–14); oral Shengxuebao (a traditional Chinese herbal formula for anemia) TID; air mattress discontinued due to T11/L2 compression fractures.	Fever worsened; pressure injury exudate increased
Day 4 of admission	Persistent respiratory symptoms; moist lung rales continue.	Initiated IV ceftriaxone 2 g once daily (April 11–17); collected wound secretion for culture; wound specialist consultation.	Antibiotic therapy optimized
Day 5 of admission	Gastric pain; fecal occult blood positive.	IV ranitidine; oral lactulose; adjusted TCM formula for Qi- and Blood-level heat.	Abdominal symptoms improved
Day 7 of admission	Wound culture result: *Escherichia coli* (sensitive to ceftriaxone). Forearm phlebitis developed.	Continued ceftriaxone; magnesium sulfate wet compress for phlebitis.	Fever resolved; systemic symptoms improved; wound exudate partially reduced.
Day 9 of admission	T11 and L2 compression fractures require further evaluation.	Orthopedic spine team consultation; decompression and long-term management plan formulated.	Stable
Day 10 of admission	CBC: WBC 2.87 × 10^9^/L↓; Neutrophils 1.55 × 10^9^/L↓; Lymphocytes 0.82 × 10^9^/L↓	Discontinued ceftriaxone.	Infection clinically controlled
Day 13 of admission (Discharge)	Cough, chest tightness, and fatigue significantly improved; vitals stable. Wound smaller with moderate drainage.	Continue oral Jieyu Xuanfei Granules (a traditional Chinese herbal formula for respiratory symptoms) and Shengxuebao; scheduled outpatient wound dressing and follow-up.	Discharged in stable condition

## 3 Nursing care headings

### 3.1 Prevention and control of respiratory infection

Decubitus pneumonia is one of the common complications in long-term bedridden patients. Studies report that the incidence of decubitus pneumonia worldwide ranges from 5.6 to 70.0% (6). In the case of this particular patient, prolonged bed confinement for 23 years due to syringomyelia has resulted in compromised overall health and severe respiratory infections. These infections are characterized by persistent fever, cough, yellow sputum, and auscultatory wet rales in the lungs. Imaging via chest CT has identified inflammation in the lower lobes of both lungs, leading to a diagnosis of bacterial pneumonia accompanied by left-sided pleural effusion. Laboratory evaluations throughout the treatment process have consistently shown elevated C-reactive protein levels, peaking at 11.71 mg/L, along with reduced albumin concentrations at 30.5 g/L and an increased erythrocyte sedimentation rate climbing to 51 mm/h. Studies advocate that respiratory management and support can effectively control infections (7, 8). The specific nursing measures are as follows (Table 2).

**Table 2:** Key points in the care of respiratory infections.

Theme	Key measures	Specific implementation
Respiratory management	Maintain airway patency	Strictly implement level I care, regularly turn over and pat the back to prevent hypostatic pneumonia, and guide the patient to practice the six-word breathing technique, primarily focusing on the “Xi” word technique. This helps expel accumulated heat from the lungs and alleviate lung congestion. During practice, guide the patient to assume a comfortable lying or semi-lying position and relax the whole body. First, inhale through the nose while simultaneously raising both arms in front of the chest, palms facing upward to shoulder height. Then, as you slowly bend your elbows and abduct your arms outward to the sides, slightly part your lips to form a thin slit. Exhale softly, slowly, and with prolonged breath, emitting a “sī” sound to expel stale air from the lungs. Upon completing the exhalation, bring your arms back in, inhale through the nose, and regulate your breath. Given the patient’s prolonged bed rest and vertebral compression fractures, the focus of exercises should be on deep, slow abdominal breathing and gentle vocalization. Limb movements must be performed slowly or with nursing assistance, repeating each exercise six times per session to avoid overexertion. This approach aims to assist in mucus clearance and prevent aspiration pneumonia.
Anti-infective therapy	Pharmacotherapy support and monitoring	Assist the physician in upgrading to intravenous infusion of ceftriaxone sodium for injection as a broad-spectrum antibiotic, at a dose of 2 g once daily, starting April 11 and continuing through April 17.
Complementary traditional Chinese medicine therapy	Clear heat, resolve phlegm, and promote lung function	Administer oral Chinese herbal formulas such as Jieji Qingre Granules (a traditional Chinese herbal formula used as adjunctive therapy for fever and respiratory symptoms) and Jieji Xuanfei Granules (a traditional Chinese herbal formula for respiratory symptoms) to clear heat, promote lung function, resolve phlegm, relieve cough, and facilitate inflammation absorption. Based on the traditional Chinese concept of medicine–food homology, foods such as pear, tremella, and white radish, which are traditionally considered beneficial for pulmonary function and sputum clearance, were incorporated into the nutritional regimen. A pear–tremella dessert prepared by the hospital nutrition department was administered as adjunctive nutritional support to promote respiratory recovery.
Respiratory support	Oxygen therapy and vital signs monitoring	Administer intermittent low-flow oxygen therapy as prescribed to support respiratory function, while closely monitoring body temperature, respiratory rate, and blood oxygen saturation.

### 3.2 Prevention and control of pressure injury infection

Upon admission assessment, a superficial second-degree ulcerated wound approximately 10 cm in diameter was visible on the patient’s back. The wound appeared reddened, was occasionally painful, and exhibited exudate. Examination of pressure ulcer secretions revealed a culture result indicating *E. coli* infection. Research indicates that malnutrition and wound infections are key factors prolonging hospital stays and increasing mortality rates among critically ill elderly patients (9). Therefore, multidisciplinary collaboration in decompression, debridement, and wound-specific management for patients with infectious pressure injuries is crucial in nursing care. The specific nursing measures are as follows (Table 3).

**Table 3:** Key points in the care of pressure injury infection.

Theme	Key measures	Specific implementation
Stress management	Decompression measures and Position management	Upon admission, an air-wave mattress was immediately implemented for pressure relief (discontinued the following day due to patient-related factors). Strict adherence to two-hourly repositioning was maintained, with soft padding used to redistribute pressure. A repositioning chart was implemented to document turning frequency and body positioning, thereby minimizing prolonged pressure on the wound.
Infection control	Wound pathogenesis monitoring and debridement	On April 11, secretions from the pressure injury site were collected for culture, confirming an *Escherichia coli* infection. Wound management was subsequently performed by a WOC nurse.
Local wound care	Combined Chinese and western medicine dressing change	Starting April 14, change dressings daily (see Table 5 for specific plan); apply Jin Di Sheng Ji ointment externally for tissue regeneration, and use a foam dressing over it to promote exudate absorption and reduce pressure.
Multidisciplinary collaboration	Consultation guidance	Invite general surgeons and WOC nurses for consultation and guidance on wound care; Invite the Spine Team from the Orthopedic Center for consultation to develop a comprehensive decompression treatment plan for vertebral compression fractures and wounds.

### 3.3 Nutritional support and complication management

At admission, the patient weighed 45 kg and was 160 cm tall, yielding a BMI of 17.58. Hemoglobin was 62.00 g/L, albumin was 30.5 g/L, and D-dimer was significantly elevated to 3,234 ng/mL, indicating hypoproteinemia, anemia, and other signs of malnutrition. Research indicates that malnutrition-related hypoproteinemia is a primary intrinsic factor contributing to pressure injuries (10) Lymphocyte percentage was 11.80%, lymphocyte count was 0.44 × 10^9^/L, C-reactive protein was 11.71 mg/L, albumin was 30.5 g/L, and erythrocyte sedimentation rate was 51 mm/h, all indicating abnormalities in infection-related indicators. Therefore, active nutritional support serves as a means to correct anemia and hypoproteinemia, while also promoting wound healing and enhancing systemic resistance to infection. The specific nursing measures are as follows (Table 4).

**Table 4:** Key points in the care of nutritional support and complication management.

Theme	Key measures	Specific implementation
Nutritional support	Correct hypoproteinemia	From April 9 to April 14, administer 10 g of human serum albumin intravenously daily to rapidly replenish protein and improve plasma colloid osmotic pressure.
Blood management	Correct severe anemia	Take Shengxuebao Compound orally three times daily to nourish the liver and kidneys, boost qi and blood production, and promote the restoration of hematopoietic function.
Gastrointestinal management	Preventing bleeding and supporting bowel movements	To manage gastric discomfort and positive fecal occult blood findings, intravenous ranitidine hydrochloride was administered for gastric mucosal protection. Manage constipation with oral lactulose solution and glycerin suppositories. Document stool color and consistency, monitor vital signs and mental status changes, and guard against potential acute gastric hemorrhage.
Management of local complications	Treatment of phlebitis	For arm phlebitis and swelling, apply wet compresses with magnesium sulfate injection to reduce local swelling and relieve pain. Monitor for local reactions and promptly change infusion sites. Select large, straight veins for the infusion pathway; choose a midline catheter if necessary.
Dietary guidance	Promote digestion and absorption	Focus on high-quality protein supplementation by increasing intake of egg whites, fish, dairy products, and soy-based foods—sources of high biological value protein—to improve nutritional status and promote wound healing. Concurrently, follow medical advice for intravenous human albumin supplementation to accelerate restoration of plasma protein levels. While ensuring adequate protein intake, instruct patients to choose light, easily digestible meals and avoid high-salt, high-fat foods. For anemia management, incorporate iron-rich foods like pork liver daily, paired with vitamin C-rich fruits to enhance iron absorption and support hematopoietic function recovery.

## 4 Discussion

This case involved a long-term bedridden patient with syringomyelia complicated by bacterial pneumonia and an infected pressure injury. Due to long-standing neurological impairment and sensory deficits, early manifestations of pressure injury were easily masked. In addition, symptoms such as fever and elevated inflammatory markers were nonspecific, and when pulmonary infection and wound infection coexisted, it was difficult to determine the primary source of infection. Furthermore, severe anemia and hypoalbuminemia impaired immune function, rendering the clinical presentation more complex. The WOC nurse identified systemic inflammatory response syndrome and pressure injury as primary nursing issues based on clinical presentation and laboratory findings. Upon admission, the patient exhibited cough, fever, anemia, hypoalbuminemia, and wound infection. Clinical care prioritized establishing a dynamic equilibrium through multidisciplinary collaboration between infection control, wound management, and nutritional support. Previous studies have also indicated that syringomyelia may be associated with delayed or progressive complications. Bauerle et al. (11) reported a case of respiratory failure in a patient with Chiari malformation–associated syringomyelia. In addition, findings by Supriya (12) and Zheng et al. (13) suggest that underlying systemic diseases or tethered cord syndrome may contribute to extensive or progressive syrinx formation. Collectively, these reports indicate that syringomyelia may evolve over a prolonged disease course, underscoring the importance of thorough etiological evaluation and long-term follow-up.

Preventing and controlling respiratory infection is crucial for reducing the risk of hypostatic pneumonia and limiting the spread of infection. Impaired cough reflexes and decreased sputum clearance due to syringomyelia predispose patients to retention of secretions and hypoventilation (14). In this case, the patient developed compression fractures of the T11 and L2 vertebrae secondary to syringomyelia and was predominantly maintained in the supine position; previous studies have shown that the supine position is associated with an increased incidence of pneumonia and pulmonary infections (3, 15). Based on the patient’s clinical condition, measures such as timed repositioning, back percussion, practicing the Six-Character Breathing Method, and focusing on the “Xu” sound exercise (Table 2) were implemented to promote sputum clearance and improve ventilation. After systematic anti-infective therapy and comprehensive respiratory management, whole-blood C-reactive protein decreased from 11.71 mg/L at admission to 5.38 mg/L, and auscultation revealed normal vocal resonance, indicating effective infection control, improved respiratory function, and promoted recovery.

The occurrence of pressure injury is closely associated with prolonged compression at the thoracolumbar junction and microcirculatory impairment. At admission, the wound was classified as superficial stage II pressure injury with a diameter of approximately 10 cm. The wound bed consisted predominantly of red granulation tissue, accompanied by exudation and local inflammatory reaction. Following deterioration with fever and infection, wound assessment by the WOC nurse revealed 60% red tissue, 30% white tissue, and 10% epithelialization, accompanied by increased exudate and rolled wound edges. Culture identified *E. coli*. Previous studies have demonstrated that epithelial formation in a moist environment occurs approximately twice as fast as in dry wounds (16), and that moist dressings help prevent surgical site infections, while hydrocolloid dressings reduce dressing change frequency in clinical practice (17). In this study, based on the theory of moist wound healing, the WOC nurse adjusted the wound treatment to foam dressing on the seventh day after admission. This approach balanced exudate management with maintaining a moist environment, supplemented by mechanical scraping to promote epithelial regeneration. After five dressing changes, reassessment showed that the wound area had decreased to 7 cm × 5 cm, epithelialization increased to 20%, and exudation was reduced, indicating that local inflammation was controlled and the process of tissue repair was gradually established. Through precise wound assessment and individualized treatment planning by the WOC nurse, pressure injury infection was effectively controlled and wound healing was promoted (Table 5).

**Table 5:** Timeline of pressure injury assessment, wound management, and outcomes.

Time	Wound images	Medical assessment	Treatment process
Admission day (Physician consultation)	None	Wound assessment: A red lesion approximately 10 cm in diameter is visible on the back, with a broken surface. The wound appears reddened, is occasionally painful, and presents with exudate.	Treatment plan: Apply Rehabilitation New Solution-soaked gauze externally (twice daily). Continue topical application of Jindi Shengji Ointment. Surgical debridement may be performed if necessary.
Fourth day of hospitalization (WOC nurse. consultation)	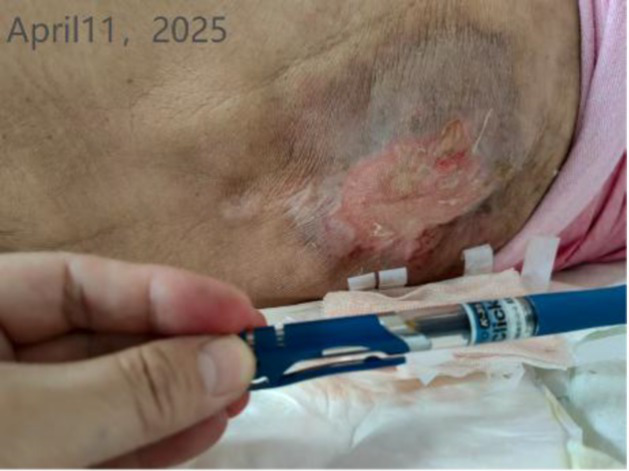	Detailed evaluation: Wound location: Thoracolumbar junction of the spine; dimensions: 10 cm × 5 cm × 0 cm; Tissue composition: Red tissue 60%, white tissue 30%, epithelialized tissue 10%. Pigmentation is present around the ulcer. Local manifestations: Increased pain, swelling at the upper right of the wound (10 cm × 8 cm × 1 cm), soft texture with fluctuance, slightly elevated skin temperature; moderate exudate, pink/red in color, odorless; NRS pain score of 3. General condition: Fever, elevated C-reactive protein, decreased albumin (hypoalbuminemia). (continued in the next column)	Diagnostic and therapeutic reflections: Analysis of the Biomechanical Mechanism Behind Recurrent Pressure Injuries: Patients with syringomyelia experience impaired voluntary movement, and prolonged bed rest leads to persistent pressure at the thoracolumbar junction of the spine. This constitutes the core factor driving recurrent pressure injuries. Evaluation and Adjustment of Preventive Measures: Although an air-filled mattress is the standard protocol for preventing pressure injuries, the patient’s family expressed concerns about its potential adverse effects on syringomyelia. Following the discussion, its use was temporarily discontinued. Instead, pressure distribution will be enhanced by increasing repositioning frequency and utilizing soft padding support. Continuity Considerations for Local Treatment Protocol: Given the patient’s prior successful treatment of pressure injuries with our hospital’s Jin Di Sheng Ji Ointment, this regimen will be maintained to ensure therapeutic continuity. Diagnosis: Stage 2 pressure injury Action plan: Wound Care: After disinfecting with povidone-iodine, wipe with saline and allow to dry. Apply Jin Di Sheng Ji ointment externally (once daily). Systemic Support: Enhance nutrition with intravenous infusion of 10 g albumin. Nursing Emphasis: Timely repositioning, Soft cushion support for pressure relief, Management of urinary incontinence (high-absorbency care products), and maintaining local cleanliness and dryness. Recommendation: Perform a color Doppler ultrasound examination of the subcutaneous swelling area. If necessary, consult with a spinal surgeon. The patient’s family has been informed of the condition’s progression and the proposed treatment plan. They have expressed understanding and will cooperate with the treatment. Close monitoring of ulcer exudate and surrounding skin changes is required. Should signs of infection emerge—such as increased redness, swelling, heat, or pain, or elevated body temperature—a complete blood count and wound exudate analysis should be promptly conducted. Antibiotic regimen adjustments may be necessary. Concurrently, the patient’s overall condition must be closely observed to maintain fluid and electrolyte balance and prevent complications.
Day 7 of hospitalization (WOC nurse, follow-up)	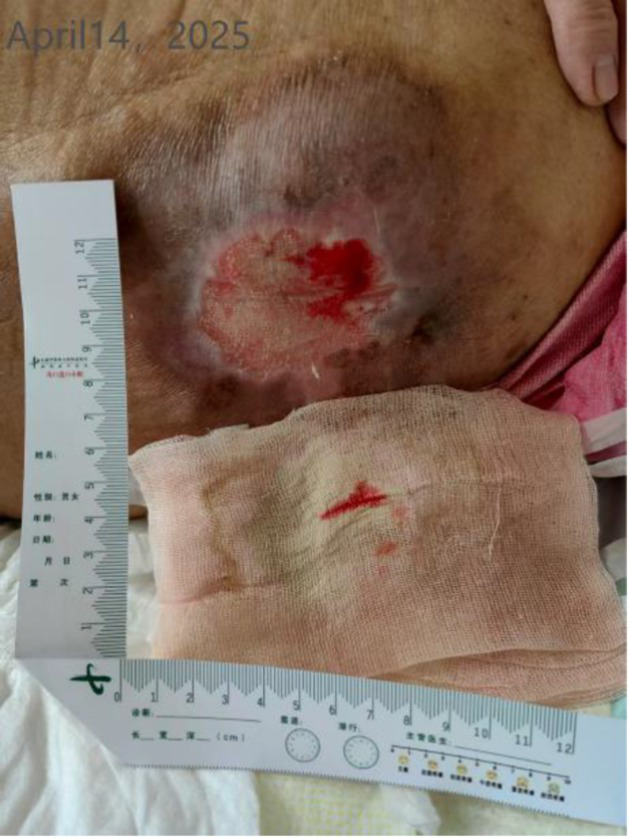	Recovery status: Jindi Shengji Ointment’s effect is not noticeable; there is a lot of exudate, requiring frequent gauze changes; there is no change in subcutaneous swelling.	Diagnostic and therapeutic reflections: Pathophysiological assessment of increased wound exudate: Moderately increased wound exudate indicates active local inflammatory response, potentially associated with tissue ischemia–reperfusion injury or underlying infection. Evidence-based rationale for treatment strategy adjustment: Based on the theory of moist wound healing, enhanced exudate management is required to prevent maceration of surrounding skin. Concurrently, the presence of wound edge curling indicates impaired epithelial migration, necessitating mechanical debridement to promote the epithelialization process. Optimizing dressing selection: Switching to foam dressings maintains a moist wound environment through high absorbency while providing pressure relief protection, aligning with international wound care guidelines. Treatment adjustment: Wound management: After disinfecting with povidone-iodine, debride the wound edges (to prevent edge curling and promote epithelialization). Continue applying Jindi Shengji Ointment externally. External dressing: Switch to foam dressing (absorbs exudate, protects the wound surface, maintains a moist environment). Recommendation: We reiterate the recommendation for a color Doppler ultrasound examination of the subcutaneous swelling and a consultation with a spine surgeon.
Day 8 of hospitalization (WOC nurse)	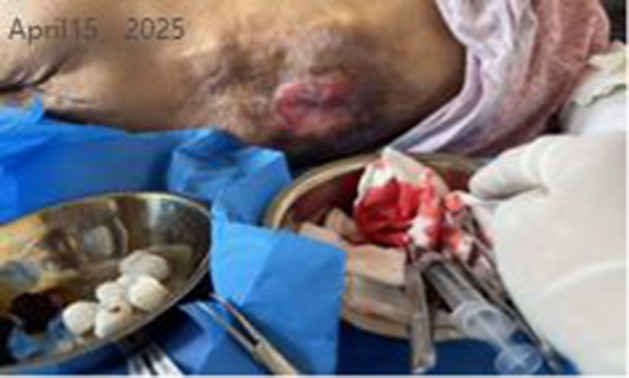	Inspection and intervention: Color Doppler ultrasound suggests inflammatory lesions cannot be ruled out. A needle aspiration was performed at the lowest point of swelling (0.5 cm depth), yielding 3 mL of fresh blood (no history of trauma).	Treatment measures: Disinfect with povidone-iodine. Insert the needle of a 5-ml syringe into the lowest point of the swelling to a depth of 0.5 cm. Fresh blood will spurt out upon needle removal. Immediately apply pressure with a gauze pad to stop bleeding. Approximately 3 mL of blood was lost. Allow the povidone-iodine to dry, then cover with a foam dressing. Diagnostic supplement: Localized hematoma (source to be determined). Recommendation: Request a consultation with a spine surgeon and perform an MRI scan to determine the extent of spinal changes.
Day 9 of hospitalization (WOC nurse)	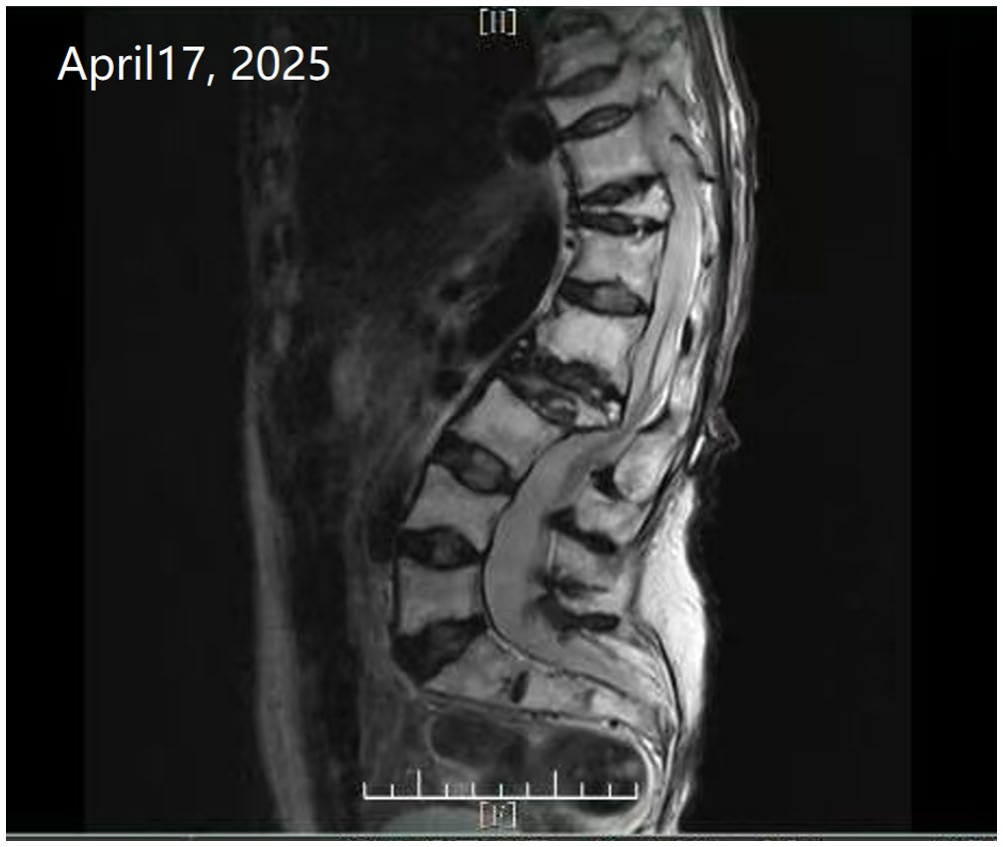	Inspection results: MRI: Lumbar degenerative changes, compression fractures of T11 and L2 vertebral bodies, disc herniation at the L1-S1 level. Spinal Surgery Consultation Findings: Change dressings regularly; If physical condition permits, surgical intervention is recommended; Actively treat the underlying disease.	After consulting multiple hospitals regarding the patient’s current condition, it has been determined that the prerequisites for surgery are not met. Follow-up plan: As the patient’s condition does not currently permit surgery, continue with foam dressing decompression and exudate collection. Avoid puncturing the hematoma for blood collection at this time (to prevent active bleeding).
Day 11 after admission (WOC nurse)	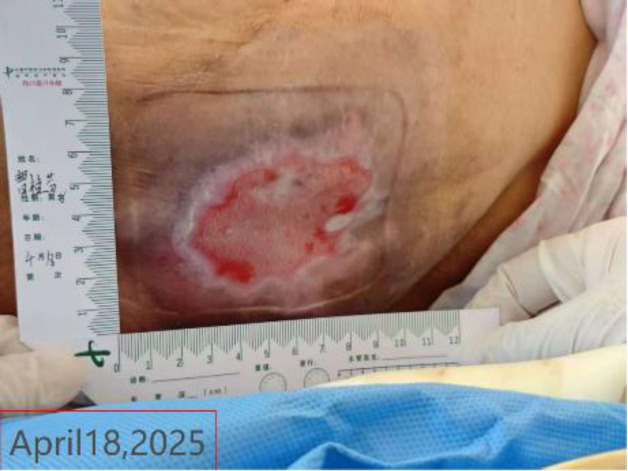 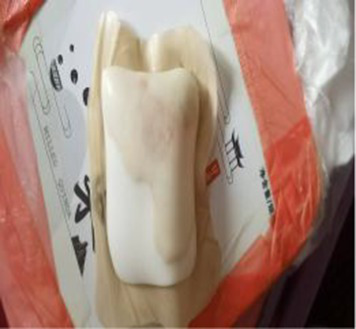	The fifth dressing change procedure involved re-evaluating the wound. Wound dimensions: Length 7 cm, Width 5 cm, Depth 0 cm Tissue type: Red 10%, White 70%, Epithelialized 20% Exudate: Odorless; Extent: Moderate; Type: Light brown Other: NRS2 points.	Disposal method as before. Disinfect with povidone-iodine. Scrape the wound bed edges with a blade to prevent curling and promote epithelialization. After povidone-iodine disinfection, wipe with saline-soaked cotton balls and allow to dry. Continue applying Jin Di Sheng Ji ointment. External dressing: Foam dressing.
The patient was discharged on hospital day 13.	None	Family feedback 2 weeks after discharge: The wound is significantly smaller than before, and the swelling has subsided. There is still some drainage, and there was a bit of bleeding today (WeChat communication).	Diagnostic and therapeutic reflections: The patient, bedridden for an extended period due to syringomyelia, experienced recurrent pressure injuries. During treatment, a subcutaneous hematoma (confirmed by aspiration) developed, accompanied by systemic conditions including hypoalbuminemia and fever. Following adjustments to local management—involving debridement, foam dressings, albumin administration, and systemic nutritional support—wound exudate decreased. Enhanced home care and follow-up are required post-discharge. Home care recommendations: Due to the caregiver’s advanced age, timely repositioning presents certain challenges. It is recommended to utilize a repositioning bed to address this issue. Continue applying foam dressings locally to reduce pressure, absorb exudate, and promote wound healing. Increase nutritional intake to prevent hypoproteinemia. Establish WeChat communication with the patient’s family for immediate contact in case of any developments.

Nutritional support was integrated throughout the entire nursing process and served as a critical component in enhancing anti-infective capacity and promoting wound healing (18, 19). On admission, the patient presented with marked hypoalbuminemia (ALB 30.5 g/L) and severe anemia (Hb 62.0 g/L); a lymphocyte percentage of 11.80% and lymphocyte count (LYM) of 0.44 × 10^9^/L indicated impaired immune function. The nutrition department and nursing staff collaborated to strengthen the intake of high-quality protein, increasing sources such as egg whites, fish, dairy products, and soy products. Simultaneously, intravenous human albumin was administered under medical guidance to rapidly restore plasma colloid osmotic pressure. To address anemia, the diet emphasized iron-rich foods such as pork liver, combined with fruits rich in vitamin C to enhance iron absorption. Dietary management followed the principles of light, easily digestible meals provided in small and frequent portions, while avoiding high-salt and high-fat foods to ensure gastrointestinal tolerance. On this basis, an individualized high-protein, high-energy, easily digestible diet was developed collaboratively by the nutrition department and nursing staff, and family members were instructed to provide small, frequent meals to maintain energy balance. Intervention effectiveness was dynamically evaluated through daily monitoring of body weight, skin turgor, and wound exudate volume. Before discharge, follow-up laboratory tests demonstrated increases in LYM (0.82 × 10^9^/L), Hb (68.0 g/L), and ALB (33.3 g/L) compared with baseline values, indicating that nutritional support effectively improved nutritional status and immune function.

Multidisciplinary collaboration played a pivotal role in this case, with physicians, nurses, nutritionists, and wound care specialists jointly developing an individualized nursing care plan to ensure continuity and effectiveness of treatment. In addition, the respiratory management, moist wound care, and individualized nutritional support adopted in this case were all routine and clinically feasible interventions that did not rely on specialized equipment, demonstrating good operability and certain potential for broader application; however, their implementation in practice should still be adjusted according to individual patient conditions. This case highlights that, for patients with complex clinical conditions, comprehensive nursing interventions should address both local and systemic management while emphasizing nutritional support and multidisciplinary collaboration to achieve optimal rehabilitation outcomes.

## 5 Conclusion

This case involved a patient with syringomyelia complicated by bacterial pneumonia and infected pressure injury, presenting a complex clinical condition that required simultaneous attention to infection control, wound management, and nutritional support. A comprehensive nursing strategy centered on respiratory management, pressure injury intervention, and individualized nutritional support was implemented, with dynamic assessment and timely adjustment under multidisciplinary collaboration among physicians, nurses, nutritionists, and wound care specialists. As a result, infection was effectively controlled, the pressure injury gradually improved, and the patient’s overall condition markedly recovered, leading to successful discharge. This case indicates that, for patients with syringomyelia accompanied by multiple complications, nursing interventions should address both local wound care and systemic management while emphasizing nutritional support and multidisciplinary collaboration to promote clinical stabilization and functional recovery. However, this study has certain limitations, including a short follow-up period and limited post-discharge continuity-of-care data, highlighting the need for further accumulation of clinical experience in future practice.

## Data Availability

The original contributions presented in the study are included in the article/supplementary material, further inquiries can be directed to the corresponding authors.
